# The G-quadruplex DNA stabilizing drug pyridostatin promotes DNA damage and downregulates transcription of Brca1 in neurons

**DOI:** 10.18632/aging.101282

**Published:** 2017-09-12

**Authors:** Jose F. Moruno-Manchon, Edward C. Koellhoffer, Jayakrishnan Gopakumar, Shashank Hambarde, Nayun Kim, Louise D. McCullough, Andrey S. Tsvetkov

**Affiliations:** ^1^ Department of Neurobiology and Anatomy, the University of Texas McGovern Medical School at Houston, TX 77030, USA; ^2^ Department of Neurology, the University of Texas McGovern Medical School at Houston, TX 77030, USA; ^3^ Summer Research Program at the University of Texas McGovern Medical School at Houston, TX 77030, USA; ^4^ Department of Microbiology and Molecular Genetics, the University of Texas McGovern Medical School at Houston, TX 77030, USA; ^5^ The University of Texas Graduate School of Biomedical Sciences, Houston, TX 77030, USA; ^6^ UTHealth Consortium on Aging, the University of Texas McGovern Medical School, Houston, TX 77030, USA

**Keywords:** G-quadruplex, DNA damage, transcription, BRCA1, neurodegeneration

## Abstract

The G-quadruplex is a non-canonical DNA secondary structure formed by four DNA strands containing multiple runs of guanines. G-quadruplexes play important roles in DNA recombination, replication, telomere maintenance, and regulation of transcription. Small molecules that stabilize the G-quadruplexes alter gene expression in cancer cells. Here, we hypothesized that the G-quadruplexes regulate transcription in neurons. We discovered that pyridostatin, a small molecule that specifically stabilizes G-quadruplex DNA complexes, induced neurotoxicity and promoted the formation of DNA double–strand breaks (DSBs) in cultured neurons. We also found that pyridostatin downregulated transcription of the *Brca1* gene, a gene that is critical for DSB repair. Importantly, in an *in vitro* gel shift assay, we discovered that an antibody specific to the G-quadruplex structure binds to a synthetic oligonucleotide, which corresponds to the first putative G-quadruplex in the *Brca1* gene promoter. Our results suggest that the G-quadruplex complexes regulate transcription in neurons. Studying the G-quadruplexes could represent a new avenue for neurodegeneration and brain aging research.

## INTRODUCTION

Understanding the mechanisms of aging is a problem of paramount importance. Brain aging is a complex phenomenon, and its mechanisms are poorly under-stood. In physiological aging, as neurons become older, they exhibit changes in synaptic plasticity, gene transcription, and DNA methylation, and are less capable of degrading oxidized material and accumulate lipofuscin. These changes are observed in the absence of significant neurological phenotypes. In unsuccessful neuronal aging, there are multiple dramatic events, including abnormally enhanced DNA damage, accumulation of impaired organelles, and protein aggregates [1, 2]. Neurons lose their synapses, processes, and degenerate. These changes are associated with neurological symptoms. It is not always clear why some age successfully and some do not [3, 4].

DNA strands containing four stretches of guanine nucleotides are able to form tetra-stranded stable secondary structures called the G-quadruplex. Although the G-quadruplexes have been studied *in vitro* for years [5], these structures are clearly important in DNA recombination, replication, telomere maintenance, and regulation of transcription [6, 7]. G-quadruplexes have been implicated in the pathogenesis of fragile X syndrome, frontotemporal dementia, and amyotrophic lateral sclerosis as the negative regulators [8]. Small molecules that stabilize the G-quadruplexes alter gene expression in cancer cells [9]. Guanine oxidation is enhanced in the genes, which are suppressed in the aged human brain [10]. Guanine oxidation increases the stability of the G-quadruplex [11]. However, how the G-quadruplexes regulate neuronal homeostasis is not clear.

Chemotherapeutic drugs, such as doxorubicin, accelerate brain aging, weaken brain neuronal networks, and induce cognitive impairments [12]. Cancer patients who underwent a doxorubicin-based therapy exhibited elevated molecular markers of senescence, such as p16INK4a and ARF [13]. We demonstrated that doxorubicin induces severe neurotoxicity and DNA double–strand breaks (DSBs) in neurons, and impairs neuronal autophagy [14, 15]. Doxorubicin intercalates into the duplex DNA, leading to the eviction of histone proteins from the chromatin [16]; however, doxorubicin can also interact with the G-quadruplex complexes, potentially stabilizing these structures [17].

In this study, we investigated if pyridostatin affects neuronal homeostasis. Pyridostatin decreased neuronal survival, damaged synapses, and promoted the formation of DNA DSBs. We found that, in neurons, pyridostatin downregulated a DNA DSB-repairing protein BRCA1 at the transcriptional level. Interesting-ly, in an *in vitro* gel shift experiment, a single–chain antibody raised against the G-quadruplex structures, binds to a synthetic oligonucleotide, which corresponds to the first putative G-quadruplex in the *Brca1* gene promoter, indicating that our data are physiologically relevant. Neuronal G-quadruplexes in general and the G-quadruplex-dependent downregulation of transcript-tion in neurons, therefore, might be a novel pathway of brain aging and a new target for mitigating age-associated neurodegeneration.

## RESULTS

### Pyridostatin is toxic for primary neurons

**Figure 1:**
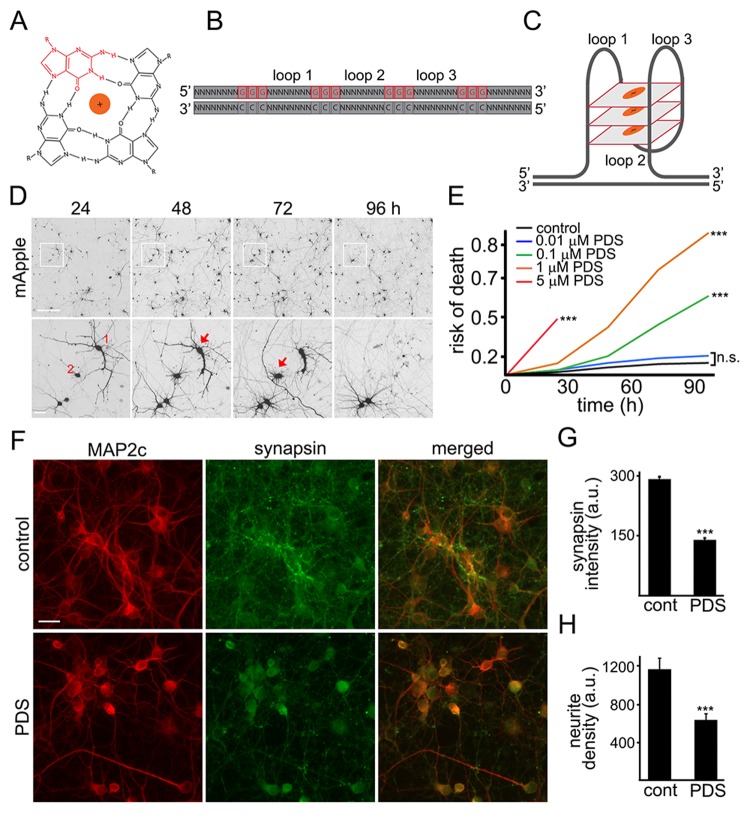
Pyridostatin is neurotoxic for primary cortical neurons (**A**) G-quadruplex is a non-canonical DNA secondary structure. Four guanine molecules (a single guanine is in red) can assemble into a square planar structure. The structure of a G-quadruplex is stabilized by hydrogen bonds between guanines and the interactions with a monovalent cation (Na^+^ or K^+^) resided in the central channel. (**B**) Repetitive guanine-rich DNA or RNA sequences have the potential to form G-quadruplex structures (**C**). (**D**) An example of survival analysis. Primary cortical neurons were transfected with mApple (a morphology and viability marker) and tracked with an automated microscope. Images collected every 24 h demonstrate the ability to return to the same field of neurons and to follow them over time. Each image is a montage of non-overlapping images captured in one well of a 24-well plate. Scale bar is 100 μm. A region from the original images at different time points is zoomed in to demonstrate longitudinal single-cell tracking (bottom panel). Red arrows depict two neurons that degenerate before 96 h after transfection. Note that neurites of the neuron 1 retract overtime. Scale bar is 20 μm. (**E**) Primary cortical neurons were transfected with mApple and treated with a vehicle or with different concentrations of pyridostatin (PDS; 0.01–5 μM). Transfected neurons were tracked with an automated microscope. Risk of death curves demonstrate that pyridostatin is neurotoxic. ***p<0.0001 (log-rank test). N.s., non-significant. Three hundred neurons were analyzed from three independent experiments. (**F**) Primary cortical neurons were treated with a vehicle (upper panel, control) or with 1 μM pyridostatin overnight (lower panel; PDS), fixed and stained with antibodies against MAP2c (red) and synapsin (green). Scale bar is 10 μm. (**G**) Quantification of the synapsin fluorescence intensity from (**F**). ***p<0.0001 (t-test). (**H**) Quantification of the neurite density from (**F**). MAP2c staining was used by the algorithm to identify and analyze neurites. ***p<0.001 (t-test). Three hundred neurons were analyzed from two independent experiments.

The G-quadruplex is a tetra-stranded secondary DNA structure formed by four stretches of guanines (Fig. 1A-C) [6]. Pyridostatin is a very selective G-quadruplex DNA-binding small molecule designed to form a complex with and stabilize G-quadruplex structures [9, 18, 19]. For cell lines, the drug is cytotoxic, and we, therefore, hypothesized that pyridostatin may be toxic for primary neurons as well. To test this, primary rat cortical cultures were transfected with the mApple construct (a red fluorescent protein). Pyridostatin or vehicle was added, and the mApple-expressing neurons were tracked for several days with an automated microscope. Loss of the red mApple fluorescence is a sensitive marker of neuronal death [20]. This method allows us to track large cohorts of individual neurons over their lifetimes and to sensitively measure their survival with statistical analyses used in clinical medicine [20]. Analyzing when each neuron lost its fluorescence allows us to measure neuronal survival with cumulative hazard statistics. By following neurons over their lifetimes, we can determine if the applied drug contributes positively, negatively, or even neutrally to neuronal fate (Fig. 1D). We found that treatment with pyridostatin substantially increased the risk of neuronal death in a dose-dependent manner (Fig. 1E). Notably, neurons exposed to pyridostatin retracted neurites before death (Fig. 1D), mimicking neuro-degenerative processes commonly observed in neurons that express ɑ-synuclein, mutant LRRK2 or mutant huntingtin [21, 22].

Neurite retraction in pyridostatin-treated neurons suggests that neurons have lost their synapses as well. Cultures were treated with pyridostatin and fixed before neurons started dying. Expectedly, we found that pyridostatin reduced the density of synapses. Pyridostatin-treated neurons also had less neurites than vehicle-treated neurons (Fig. 1F-H).

### Pyridostatin induces DNA DSBs

**Figure 2:**
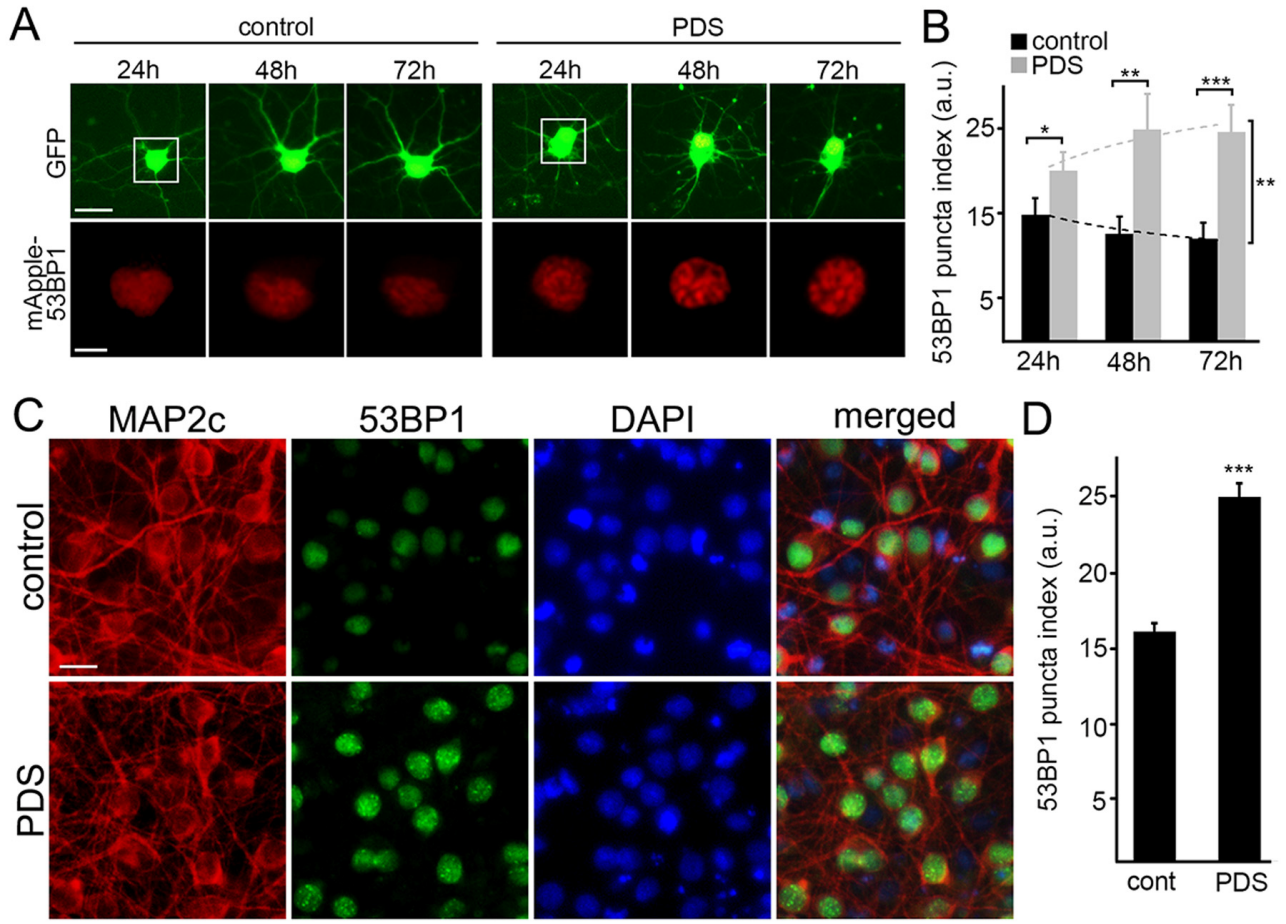
Pyridostatin promotes the formation of 53BP1-positive puncta in primary neurons (**A**) Primary cortical neurons were transfected with GFP and mApple-53BP1trunc constructs, and then treated with a vehicle (left panel; control) or with 1 μM pyridostatin (right panel; PDS). Neurons were imaged with an automated microscope every 24 h for 3 days. Scale bar is 5 μm. (**B**) Quantification of the mApple-53BP1trunc puncta index from (**A**) at different times. The puncta index was estimated by measuring the standard deviation of the 53BP1 fluorescence intensity. Note that 53BP1 puncta index is higher in pyridostatin-treated neurons than control neurons. *p<0.01, **p<0.001, and ***p<0.0001 (t-test). A.u., arbitrary units. Two hundred neurons were analyzed from two independent experiments. (**C**) Primary cortical neurons were treated with a vehicle (upper panel; control) or with 1 μM pyridostatin (bottom panel; PDS) overnight, fixed, and stained for MAP2c (red), a marker of DNA damage 53BP1 (green), and with the nuclear Hoechst dye (blue). Scale bar is 10 μm. (**D**) Quantification of the 53BP1 puncta index from (**C**). Pyridostatin (PDS) increased the 53BP1 puncta index compared to control neurons (cont). ***p<0.0001 (t-test). A.u., arbitrary units. Three hundred neurons were analyzed from three independent experiments.

Putative G-quadruplexes are present on average once per 10 kb of the human genome [23]. In addition to inhibiting DNA replication, the pyridostatin–DNA complex may stall DNA polymerase during transcription [19, 24]. DNA damage may then occur via the action of endonucleases, through a mechanism of so-called transcription–coupled–repair poisoning [25]. Since pyridostatin induces DNA DSBs in cell lines [9], we hypothesized that pyridostatin may induce DNA damage in primary neurons. To analyze DNA damage in neurons in our lab, we recapitulated a recent single–cell analysis technique based on the expression of a truncated 53BP1 DSB reporter in cell lines [26]. The mApple-53BP1trunc construct was expressed in two cohorts of primary neurons along with GFP, a marker of cell viability and morphology. The first neuronal cohort was treated with a vehicle; the second cohort was treated with pyridostatin. Both neuronal cohorts were then followed longitudinally (Fig. 2A). Expectedly, the mApple-53BP1trunc construct formed puncta in neurons exposed to pyridostatin, reflecting DNA damage (Fig. 2B).

Since overexpressed proteins sometimes aggregate in primary neurons, we decided to confirm our finding with the endogenous 53BP1 protein. Cultured neurons were treated with pyridostatin, fixed and stained for endogenous 53BP1 and MAP2c, and with the nuclear DAPI dye. Neurons exposed to pyridostatin had punctuated structures of 53BP1 localized to their nuclei (Fig. 2C,D).

**Figure 3:**
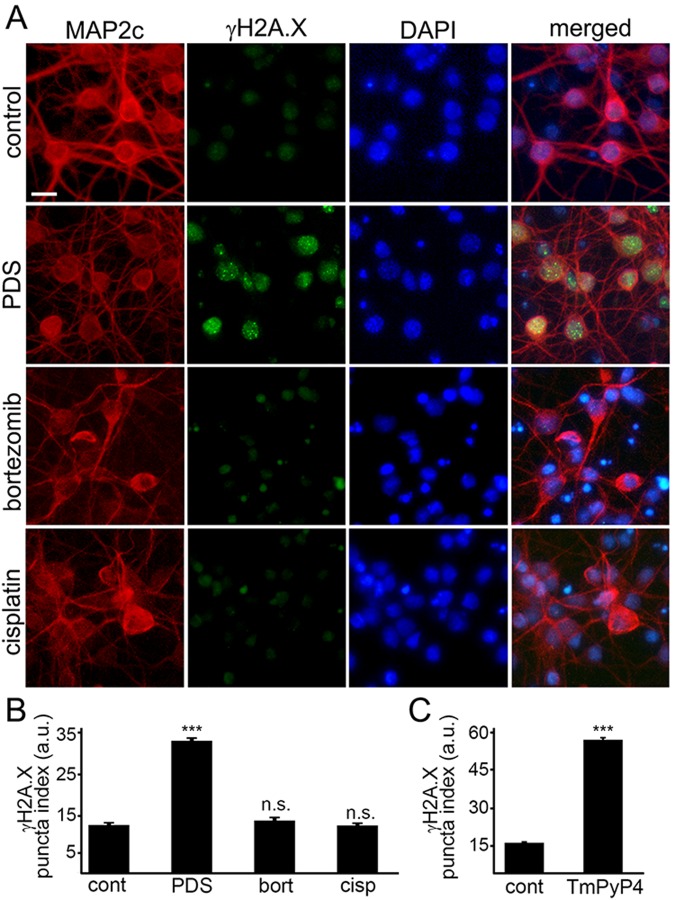
G-quadruplex-stabilizing drugs upregulate γH2A.X (**A**) Primary cortical neurons were treated with a vehicle (control) or with 1 μM pyridostatin (PDS) or with 200 nM bortezomib (bortezomib) or with 10 μM cisplatin (cisplatin) overnight, fixed, and stained for MAP2c (red), a marker of DNA DSBs phosphorylated histone H2A variant X, γH2A.X (green), and with the nuclear Hoechst dye (blue). Scale bar is 10 μm. (**B**) The puncta index was estimated by measuring the standard deviation of the γH2A.X fluorescence intensity. Primary cortical neurons were treated with a vehicle (control, cont) or with 1 μM pyridostatin (PDS), or with 200 nM bortezomib (bort), or with 10 μM cisplatin (cisp) overnight, then fixed, and immunostained with antibodies against MAP2c and γH2A.X, and co-stained with the nuclear Hoechst dye (blue). ***p<0.0001 (t-test). N.s., non-significant (p_bort_=0.1995; p_cis_=0.8228). A.u., arbitrary units. Four hundred neurons were analyzed from three independent experiments. (**C**) Primary cortical neurons were treated with a vehicle (control, cont) or with a G-quadruplex stabilizing drug, TmPyP4 (1 μM, overnight), then fixed, immunostained against MAP2c and γH2A.X, and co-stained with the nuclear Hoechst dye (blue). ***p<0.0001 (t-test). A.u., arbitrary units. Four hundred neurons were analyzed from three independent experiments.

Phosphorylated histone H2A variant X (γH2A.X) is commonly used as a read-out of DNA DBSs [14]. Therefore, to make sure that γH2A.X is phosphorylated when neurons are treated with pyridostatin, as seen in non-neuronal cells, neuronal cultures were treated with pyridostatin, fixed and stained for γH2A.X and MAP2c, and with the nuclear DAPI dye. Neurons exposed to pyridostatin had more staining of γH2A.X in the neuronal nuclei than control neurons (Fig. 3A,B). Our data suggest that stabilized G-quadruplexes contribute to DNA damage in neuronal cells.

Since DNA damage may, in principle, be a general phenomenon in degenerating neurons, we determined if other neurotoxic small molecules, bortezomib and cisplatin, promote DNA DSBs. Bortezomib, a chemo-therapy drug that inhibits the ubiquitin-proteasome system (UPS), was used to promote neurodegeneration. Inhibitors of the UPS are highly neurotoxic [27]. Cisplatin, another anti-tumor agent, cross-links DNA and, therefore, promotes cytotoxicity [28]. Cisplatin is also highly toxic for neurons [29]. Primary neurons were treated with bortezomib and cisplatin, and then fixed and stained for γH2A.X and MAP2c. Neither bortezomib nor cisplatin induced DNA DSBs (Fig. 3A,B). Our data demonstrate that DNA DSBs are not a general effect induced by cytotoxic agents, and pyridostatin-associated DNA damage likely depends on pyridostatin's G-quadruplex-stabilizing properties.

Do other G-quadruplex-stabilizing drugs induce DNA DSBs? We tested if TmPyP4 [30], a cationic porphyrin, induces accumulation of γH2A.X-positive puncta in cultured primary neurons. Neuronal cultures were treated with TmPyP4 under conditions identical to the pyridostatin experiment, fixed and stained for γH2A.X and MAP2c, and with the nuclear DAPI dye. TmPyP4 stimulated the formation of γH2A.X-positive puncta in neurons (Fig. 3C).

### Pyridostatin downregulates BRCA1 in neurons

The breast cancer type 1 susceptibility protein, BRCA1, repairs DNA DSBs in non-neuronal cells and neurons. In addition to well-studied BRCA1 dysregulation in cancer cells, BRCA1 protein is downregulated in the brains of patients with Alzheimer's disease. Amyloid-beta, a peptide associated with Alzheimer's disease, downregulates BRCA1 leading to the formation of DNA DSBs [31].

**Figure 4:**
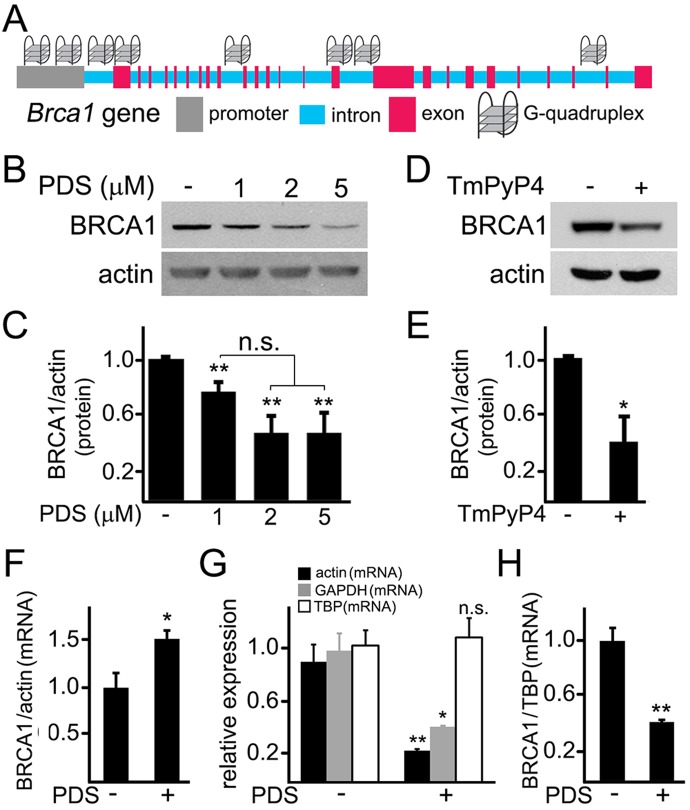
Pyridostatin downregulates BRCA1 levels in primary neurons (**A**) A scheme of the *Brca1* gene and its promoter showing putative G-quadruplex sequences. (**B**) Primary neurons were treated with a vehicle (control, -) or with different concentrations of pyridostatin (PDS; 1, 2 or 5 μM) overnight. Then neurons were collected, lysed, and samples were processed with western blotting. Note that pyridostatin reduced BRCA1 protein levels in a dose-dependent manner. Actin was used as a loading control. (**C**) Quantification of BRCA1 protein levels normalized to actin from (**B**). **p_(cont-1 μM)_=0.005, **p_(cont-2 μM)_=0.0015, **p_(cont-5 μM)_=0.0043 (ANOVA). N.s., non-significant, p_(1 μ M−2 μM)_=0.0547, p_(1 μM−5 μM)_=0.0854. Results were pooled from five independent experiments. (**D**) Primary neurons were treated with a vehicle (control, -) or with a G-quadruplex stabilizing drug, TmPyP4 (1 μM), overnight. Neurons were lysed, and samples were processed by western blotting. TmPyP4 reduced BRCA1 protein levels. Actin was used as a loading control. (**E**) Quantification of BRCA1 protein normalized to actin from (**D**). *p<0.0276 (t-test). Results were pooled from four independent experiments. (**F**) Primary neurons were treated with a vehicle (control; -) or with 2 μM pyridostatin (PDS; +) overnight. The expression of *Brca1* was determined by qRT-PCR relative to *Actin*. *p=0.0445 (t-test). Results were pooled from three independent experiments. (**G**) Primary neurons were treated with a vehicle (control; -) or with 2 μM pyridostatin (PDS; +) overnight. Relative expression of the *Actin*, *Gapdh* and *Tbp* genes was determined by qRT-PCR. *p=0.0112, **p=0.0039 (t-test). N.s., non-significant (p=0.7583). Results were pooled from three independent experiments. (H) Primary neurons were treated with a vehicle (control; -) or with 2 μM pyridostatin (PDS; +) overnight. The expression of *Brca1* was determined by qRT-PCR normalized to *Tbp.* **p=0.0033 (t-test). Results were pooled from three independent reactions.

The *Brca1* gene in *Rattus norvegicus* is a long gene that has two known alternatively spliced RNA products. We hypothesized that the *Brca1* gene may contain putative G-quadruplexes. We analyzed the rat *Brca1* gene sequence with the QGRP (putative quadruplex forming G-rich sequences) mapper and discovered that, in the gene, two putative sequences in the coding sequence can arrange into the G-quadruplexes. Additionally, its introns contain four putative G-quadruplex-forming sequences. There are also two putative G-quadruplex-forming sequences in the *Brca1* gene promoter (Fig. 4A). Therefore, pyridostatin may stabilize these putative G-quadruplexes and inhibit the *Brca1* gene transcription.

First, we tested if the levels of the BRCA1 protein are changed in neurons exposed to pyridostatin. Cultured cortical neurons were treated with pyridostatin, and then their extracts were analyzed by western blotting. Remarkably, the levels of the BRCA1 protein were indeed decreased (Fig. 4B,C). Levels of actin in samples were not affected, suggesting that the actin protein itself is not a target of pyridostatin. To confirm this finding, we investigated if TmPyP4 reduces the levels of BRCA1 in neurons. The BRCA1 protein levels were downregulated in TmPyP4-treated neurons as well (Fig. 4D,E). We, therefore, conclude that, in primary neurons, stabilizing G-quadruplex DNA downregulates BRCA1, at least at the protein level. As a result, DNA DSBs can accumulate due to the action of nucleases and to downregulated BRCA1, at least in part.

Pyridostatin was designed to selectively bind to G-quadruplex DNA and to stabilize the G-quadruplex structures [9, 18, 19]. Nevertheless, the drug can theoretically inhibit protein translation or even affect its degradation. To test whether transcription of the *Brca1* gene is directly affected, we sought to investigate whether pyridostatin changes BRCA1's mRNA levels in neuronal cells exposed to the drug. Neuronal cultures were treated with pyridostatin; mRNA was extracted and analyzed. Remarkably, the levels of BRCA1 mRNA were higher in pyridostatin-treated neurons, when normalized to the control actin mRNA (Fig. 4F).

An increase in normalized BRCA1 mRNA levels was unexpected, but could reflect a change in the levels of actin mRNA itself in pyridostatin-treated neurons. To test that, we measured the levels of actin mRNA in vehicle-treated neurons and in neurons exposed to pyridostatin, and discovered that, indeed, actin mRNA was down-regulated, indicating that pyridostatin targets the *Actin* gene as well. Interestingly, we did not observe a decrease in the levels of the actin protein (Fig. 4B), which indicates that actin is a long-lived protein.

Searching for other loading controls, we tested mRNA levels of glyceraldehyde 3-phosphate dehydrogenase (GAPDH) and TATA-binding protein (TBP). Interestingly, neurons exposed to pyridostatin had reduced levels of GAPDH, but the levels of TBP mRNA remained unchanged (Fig. 4G). Therefore, TBP mRNA was used as loading control, and in this case, BRCA1 mRNA was downregulated in neurons treated to pyridostatin (Fig. 4H).

We were surprised that pyridostatin downregulated BRCA1, actin, and GAPDH, but not TBP mRNAs. We, therefore, analyzed the sequences of the *Actin*, *Gapdh* and *Tbp* genes and their promoters with the QGRP mapper. Both *Actin*'s and *Gapdh*'s promoters contain two putative G-quadruplex-forming sequences, and *Actin* contains one putative G-quadruplex sequence. Remarkably, neither *Tbp* nor its promoter contains a putative G-quadruplex-forming sequence, indicating that our data generated with pyridostatin are physiologically relevant. Future studies will identify more genes regulated by the G-quadruplex.

### Overexpressed BRCA1 mitigates pyridostatin-induced DNA damage

**Figure 5:**
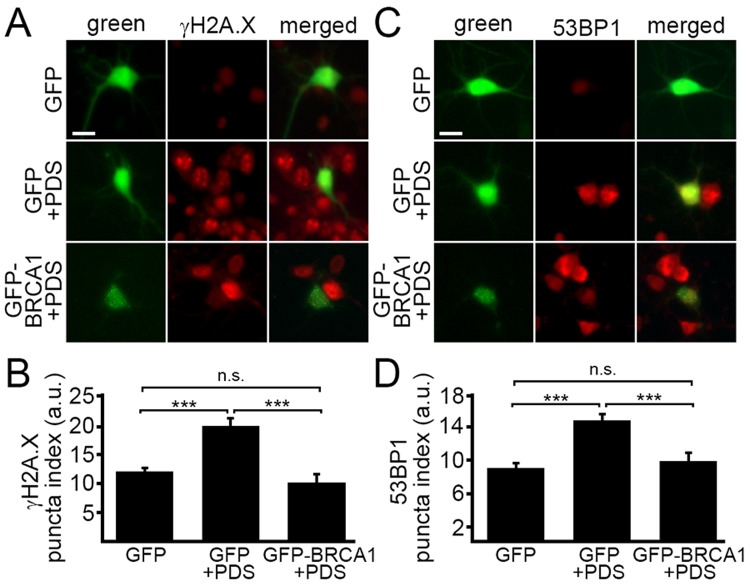
Ectopically expressed BRCA1 mitigates DNA damage associated with pyridostatin treatment (**A**) Primary cortical neurons were transfected with GFP or with GFP-BRCA1 constructs, and then treated with a vehicle (control, GFP) or with 1 μM pyridostatin (GFP+PDS and GFP-BRCA1+PDS), overnight, fixed, and stained for a marker of DNA DSBs phosphorylated histone H2A variant X, γH2A.X (red). Scale bar is 10 μm. (**B**) The puncta index was estimated by measuring the standard deviation of the γH2A.X fluorescence intensity from (**A**). ***p<0.0001 (ANOVA). N.s., non-significant, p=0.243. A.u., arbitrary units. Two hundred neurons were analyzed from two independent experiments. (**C**) Primary cortical neurons were transfected with GFP or with GFP-BRCA1 constructs, and then treated with a vehicle (control, GFP) or with 1 μM pyridostatin (GFP+PDS, GFP-BRCA1+PDS), overnight, fixed, and stained for a marker of DNA DSBs 53BP1 (red). Scale bar is 10 μm. (**D**) The puncta index was estimated by measuring the standard deviation of the γH2A.X fluorescence intensity from (**C**). ***p<0.0001 (ANOVA). N.s., non-significant, p=0.441. A.u., arbitrary units. Two hundred neurons were analyzed from two independent experiments.

BRCA1 is required for DNA damage repair. We wondered if ectopically increasing BRCA1 protein levels would attenuate DNA damage associated with the exposure to pyridostatin. To test this, neurons were transfected with a plasmid that encodes GFP or GFP-BRCA1. Neurons were treated with a vehicle or with pyridostatin overnight and then fixed and immuno-stained with antibodies against γH2A.X or 53BP1 (Fig. 5A–D). The γH2A.X and 53BP1 puncta indexes in GFP-BRCA1-expressing neurons treated with pyridostatin were lower than in GFP-expressing neurons exposed to pyridostatin (Fig 5A–D). These results indicate that ectopically expressed BRCA1 mitigates DNA damage induced by pyridostatin treatment in neurons and demonstrate that BRCA1 is critical for maintaining neuronal genome integrity.

### The BG4 antibody binds to a synthetic oligonucleotide that corresponds to a putative G-quadruplex in the *Brca1* gene promoter

A single–chain BG4 antibody was identified through a phage display method with a library of 2.3 x 10^10^ different single–chain antibody clones with an *in vitro* selection for G-quadruplex structures. The BG4 antibody recognizes intramolecular and intermolecular DNA G-quadruplexes with a nanomolar affinity and does not bind to single-stranded or double-stranded DNA [32]. To determine if the putative G-quadruplexes folds into a four-strand structure that can be recognized by the BG4 antibody, we synthesized three Cy5-labeled oligonucleotides: the BRCA1-G1 and BRCA1-G2 oligonucleotide corresponded to the two putative G-quadruplex sequences in the *Brca1* promoter, and the BRCA1-cont was a control oligonucleotide, which cannot fold into a G-quadruplex structure.

The G-quadruplexes form a stable structure in the presence of K^+^ and Na^+^ ions and do not properly fold into a G-quadruplex in a buffer that contains Li^+^ [33].

**Figure 6:**
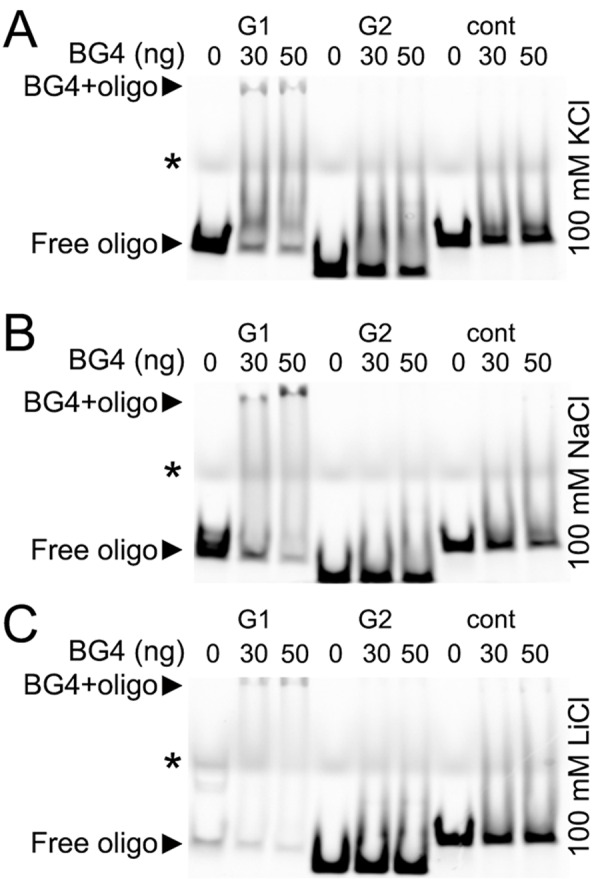
The G-quadruplex-specific antibody BG4 binds to a G-rich oligonucleotide derived from the promoter of *Brca1* (**A**) The Cy5-conjugated BRCA1-G1 (G1), BRCA1-G2 (G2), and BRCA1-cont (cont) oligonucleotides (see Methods) were heat-denatured and then slow-cooled in the presence of K^+^ (KCl) to allow the formation of a secondary structure. 1.5 pmoles of each oligonucleotides (oligo) and 0 (a buffer alone), 30 or 50 ng of the BG4 antibody were incubated in a buffer, which contained 100 mM KCl. Note the bands that correspond to the free BRCA1-G1 (at the bottom) and the BRCA1-G1 bound to the BG4 antibody (at the gel top), both indicated with arrow heads. An asterisk marks the faint bands, which resulted from a loading dye. (**B**) The Cy5-conjugated BRCA1-G1 (G1), BRCA1-G2 (G2), and BRCA1-cont (cont) oligonucleotides were prepared in the presence of Na^+^ (NaCl). 1.5 pmoles of each oligonucleotides (oligo) and 0 (a buffer alone), 30 or 50 ng of the BG4 antibody were incubated in a buffer, which contained 100 mM NaCl. Note the bands that correspond to the free BRCA1-G1 (at the bottom) and the BRCA1-G1/BG4 complex (at the gel top), depictured with arrow heads. An asterisk marks the loading dye bands. (**C**) The Cy5-labeled BRCA1-G1 (G1), BRCA1-G2 (G2), and BRCA1-cont (cont) oligonucleotides were prepared in the presence of Li^+^ (LiCl). 1.5 pmoles of each oligonucleotides (oligo) and 0 (a buffer alone), 30 or 50 ng of the BG4 antibody were incubated in a buffer, which contained 100 mM LiCl. Note the bands that correspond to the free BRCA1-G1 (at the bottom) and a weak BRCA1-G1/BG4 complex (at the gel top), depictured with arrow heads. An asterisk marks the loading dye bands.

We incubated the BG4 antibody with the BRCA1-G1 oligonucleotide, the BRCA1-G2 oligo-nucleotide, and the BRCA1-cont oligonucleotide in the buffers that contained K^+^, Na^+^, or Li^+^ ions (Fig. 6A–C). The BG4 antibody did not form a complex with the BRCA1-cont oligonucleotide in all conditions tested (Fig. 6A–C). The BRCA1-G2 oligonucleotide also did not form a complex with the BG4 antibody. However, the BG4 antibody did bind to the BRCA1-G1 oligo-nucleotide, which was reflected by the slower migrating band on the gel, in both K^+^ and Na^+^ buffers. In the presence of Li^+^, the BRCA1-G1 oligonucleotide formed a weaker complex with the BG4 antibody (Fig. 6A–C).

We were surprised that the BG4 antibody recognized the BRCA1-G1 oligonucleotide as a G-quadruplex structure, but did not bind to the BRCA1-G2 oligonucleotide. In fact, however, the BRCA1-G1 oligonucleotide (AGGGCTCTCCCTTGGGGGGGGGGGGCAGGAAGGGA) contains more guanines and longer guanine–runs than the BRCA1-G2 oligo-nucleotide (AGGGAGGCCATGGGACGGGAAGACTTGGGT), indicating that the G-quadruplex formed by the BRCA1-G1 oligonucleotide may be significantly more stable.

## DISCUSSION

**Figure 7:**
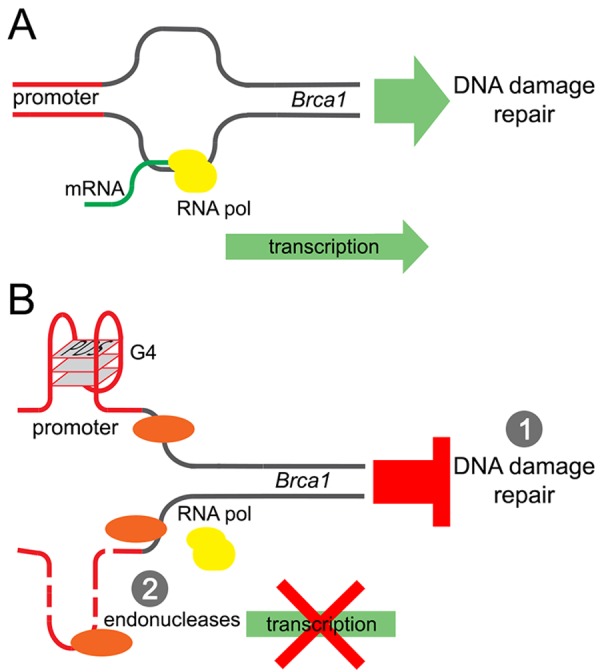
A model of the *Brca1* gene downregulation by pyridostatin (**A**) *T*ranscription of the *Brca1* gene in primary neurons under basal conditions. (**B**) Pyridostatin binds to and stabilizes the G-quadruplex structures present in the *Brca1* gene promoter sequence. The pyridostatin/DNA complex stalls DNA polymerase during transcription and downregulates *Brca1* gene expression. (1) *Brca1* downregulation may then hinder DNA damage repair and DNA DSBs accumulate as a result. (2) In addition, DNA damage may occur due the action of endo-nucleases through a mechanism of transcription–coupled–repair poisoning [25].

Our results revealed a potentially new mechanism of neurodegeneration and transcriptional regulation in neurons (Fig. 7). Pyridostatin, a G-quadruplex-stabilizing small molecule, causes neurite retraction, synaptic loss, and dose-dependent neuronal death. In cultured primary neurons, pyridostatin induces the formation of DNA DSBs. Remarkably, the drug downregulates the BRCA1 protein, a protein that guards and repairs the neuronal genome, at the transcriptional level. Using an *in vitro* gel shift experiment, we demonstrated that an antibody developed against the G-quadruplex structures binds to a synthetic oligo-nucleotide, which was synthesized to correspond to the first putative G-quadruplex sequence in the *Brca1* gene promoter. Our results suggest that small molecules with pyridostatin-like properties promote neurodegeneration and identify a novel mechanism of transcriptional regulation in neurons.

Up to 700,000 G-quadruplex-induced polymerase-stalling sites were detected in the human genome with a high-throughput sequencing method [34]. In mammalian cells, the putative G-quadruplex structures are frequent in oncogenes and regulatory genes [35, 36]. The G-quadruplex in the promoter region negatively regulates the expression of the oncogenes c-MYC and KRAS. Small molecules that stabilize the G-quadruplex structures significantly decrease c-MYC and KRAS expression [37, 38]. Notably, the G-quadruplex structures positioned as far as 3,500 base pairs away from the transcription start codon reduce gene expression [39]. An antibody-based chromatin immunoprecipitation and high-throughput sequencing revealed about 10,000 G-quadruplex structures in the chromatin of human epidermal keratinocyte cells, mostly in nucleosome-free regions [40]. Our data suggest that neuronal cells have a neuron-specific set of “active” G-quadruplex structures. In the aged brain, in which G-quadruplex-stabilizing guanine oxidation is frequent, many genes involved in synaptic plasticity, vesicular transport, mitochondrial function, and protein turnover are downregulated [10]. Importantly, several genes involved in learning and memory have been studied with a great interest and are downregulated in aged brains. These are such as *Egr1* and *Arc*, which whose promoters are abnormally methylated during brain aging [41-43]. The levels of synaptic plasticity-related *Agrn*, *Gap43*, *Syn1* and *Vgf* mRNAs also go down during aging [44, 45]. All these genes or their promoters contain putative G-quadruplexes.

Interestingly, topotecan, an inhibitor of topoisomerase 1, which resolves DNA supercoiling during replication and transcription, downregulates the expression of a large number of long genes, including many neuronal genes [46, 47]. The gene's length remarkably predicts whether inhibiting topoisomerase 1 downregulates gene transcription. Maab *et al*. speculate that topoisomerase 1 is the “Achilles heel” of neurons because its inhibition downregulates a number of long genes, which are critical for proper neuronal function [46]. Our data suggest that the G-quadruplex is the “chink in the armor” of neurons. The presence of a G-quadruplex may predict if a gene is sensitive to a potential G-quadruplex stabilizer. Intriguingly, there is a group of genetic disorders caused by mutations in the genes encoding DNA helicases, the enzymes that unwind DNA during replication and transcription [48]. Significantly, the G-quadruplexes are targeted by several DNA helicases, and mutations in these enzymes are associated with Cockayne syndrome, a rare fatal disease with a developmental and neurodegenerative phenotype [49-51].

In summary, we demonstrated that pyridostatin induces neurodegeneration, promotes DNA DSBs, and downregulates transcription in neurons. Our results are in an agreement with previous findings that stabilizing the G-quadruplexes is cytotoxic. Future studies in mice will determine if the G-quadruplex structures contribute to brain aging and neurodegeneration.

## METHODS

### Chemicals and plasmids

Pyridostatin (PDS) was obtained from Cayman Chemical (#18013). TmPyP4 was from Calbiochem (#613560). Hoechst dye was from Santa Cruz Biotechnology (#sc-394039). Antibodies against synapsin were from Synaptic Systems (#106002). Antibodies against BRCA1 (#ab191042) and 53BP1 (#ab172580) were from Abcam. Antibodies against γH2A.X (JBW301) were from EMD Millipore (#05636). Rabbit antibodies against MAP2c (H-300, #sc-20172), and mouse antibodies against MAP2c (A-4, #sc-74421) were from Santa Cruz Biotechnology. Antibodies against β-actin (8H10D10) were from Cell Signaling (#3700). Anti-rabbit Alexa Fluor 488–labeled (#A11008), anti-mouse Alexa Fluor 488–labeled (#A11001), anti-rabbit Alexa Fluor 546–labeled (A11010), and anti-mouse Alexa Fluor 546-labeled (#A11003) secondary antibodies were from Life Technologies. pCAG-mApple-hTP53BP1 (1220-1709aa) was synthesized by Vectorbuilder. pSANG10-3F-BG4 was from Addgene (#55756; deposited by Dr. Shankar Balasubramanian, the University of Cambridge). pDEST-FRT/T0-GFP-BRCA1 was from Addgene (#71116; deposited by Dr. Durocher, the University of Toronto).

### Neuronal and astrocytic cultures and transfection

Primary cortical neurons and astrocytes were isolated from rat embryos (E17-E19), and plated on 24-well tissue-culture plates (4x10^5^/well) coated with poly-D-lysine (BD Biosciences), as described [14, 52]. Neuronal cultures were maintained in Neurobasal Medium (Life Technologies) supplemented with B-27 (Life Technologies), GlutaMAX (Life Technologies), and penicillin-streptomycin (Life Technologies). Some primary cultures were transfected after 4 days *in vitro* with Lipofectamine 2000 (Invitrogen) and with a total of 1 μg of plasmid DNA per well, as described [14, 52-54].

### Fluorescence microscopy and image analysis

Live cell imaging was performed with the EVOS microscopy system (Life Technologies). Fixed and immunostained cells were imaged with the RFP (MAP2c, 53BP1 and γH2A.X) filter, the GFP (synapsin, 53BP1, γH2A.X and GFP-BRCA1) filter, and the DAPI filter (Hoechst). γH2A.X and 53BP1 fluorescence was analyzed by the puncta index, which is the standard deviation of the intensities measured among pixels within the neuronal nuclei. Low puncta index represents diffuse localization, whereas a high puncta index represents punctate localization.

### Longitudinal fluorescent microscopy and survival analysis

For longitudinal imaging of DNA damage in living cells, primary cortical neurons were transfected with pCAG-mApple-hTP53BP1 (mApple-53BP1trunc) and pGW1-GFP, and then treated with a vehicle or 1 μM PDS. For survival experiments, primary cortical neurons were transfected with pGW1-mApple and treated with a vehicle or different concentrations of PDS (0.01, 0.1, 1 and 5 μM). Then, cultures were imaged every 24 h for 5 days. The plate was placed on the microscope stage, which automatically moves the 20x objective to the center of the first well and collects fluorescence images with the red filter (mApple, mApple-53BP1trunc) and the green filter (GFP), thereafter moving the stage to each adjacent field in the well. These steps are repeated until all required wells are imaged. For tracking the same group of neurons over time, an image of the fiduciary field with neurons on the plate was collected at the first time-point and used as a reference image. Each time the same plate was imaged thereafter, the fiduciary image was aligned with the reference image. Neurons that died during the imaging interval were assigned a survival time. These event times were used to obtain the exponential cumulative survival graphs and analyzed for statistical significance by Log-Rank test with JMP software (SAS Institute) as described [14, 54, 55].

### Immunocytochemistry

Primary cortical neurons were fixed with 4% paraformaldehyde for 15 min at room temperature. Then, neurons were permeabilized with 0.1% Triton X-100 in PBS, and blocked with 1% bovine serum albumin in PBS for 1 h at room temperature. Cells were incubated with a primary antibody (anti-MAP2c, anti-synapsin, anti-53BP1, or anti-γH2A.X) diluted in blocking buffer at 4°C overnight. Cells were washed, and incubated with a secondary antibody (anti-rabbit-Alexa Fluor 546, anti-mouse-Alexa Fluor 546, anti-mouse-Alexa Fluor 488, anti-rabbit-Alexa Fluor 488) diluted in blocking buffer for 1 h at room temperature. Nuclei were stained with Hoechst dye in PBS for 2 min. Cells were washed and analyzed.

### Immunoblotting

Neuronal cultures were collected and lysed in RIPA buffer (150 mM NaCl, 1% Nonidet P40, 0.5% sodium deoxycholate, 0.1% SDS and 50 mM Tris/HCl (pH 8.0), with phosphatase and protease inhibitors cocktail) on ice. Lysates were vortexed and cleared by centrifugation (14000 *g*, 10 min, 4°C). Supernatants were collected, and protein concentrations were determined by the Bicinchoninic Acid Protein Assay Kit (Thermo Scientific), according to the manufacturer's protocol. Samples were analyzed by SDS/PAGE (4-12% gradient gels), and proteins were transferred on to nitrocellulose membranes using the iBlot2 system (Life Technologies). Membranes were blocked with 5% (w/v) non-fat dried skimmed milk for 1 h at room tempera-ture, and they were incubated with anti-BRCA1 and anti-actin, as a loading control, overnight at 4°C. Membranes were then washed with TBS (Tris-buffered saline; 10 mM Tris/HCl and 150 mM NaCl (pH 7.4)) and incubated with anti-rabbit-HRP and anti-mouse-HRP for 1 h at room temperature. Chemiluminescent signal was visualized with Prometheus ProSignal Pico (Genesee Scientific) on Blue Devil autoradiography films (Genesee Scientific).

### RNA extraction and qRT-PCR

Total RNA was extracted from primary culture using the RNeasy Mini kit (#74104, Qiagen), and then reverse transcribed using iScript Reverse Transcription SuperMix (#1708840, Bio-Rad), according to the manufacturer's protocol. RT-qPCR was performed using a Bio-Rad CFX96 Touch machine using SSoAdvanced Universal SYBR Green (#1725275, Bio-Rad) for visualization and quantification according to the manufacturer's instructions. Primer sequences were: BRCA1, forward: 5′-GCAGATGGGCTGACAGTAAA-3′, reverse: 5′ -GCTTTCTACCACAGAGGGAATC-3′, TBP, forward: 5′ -AGTGCCCAGCATCACTGTTT-3′, reverse: 5′ -GGTCCATGACTCTCACTTTCTT-3′. β-actin, forward: 5′ -CTTCACCACCACGGC-3′, reverse: 5′ -CCATCTCTTGCTCGAAG-3’. GAPDH, forward: 5′ -CAACTACATGGTCTACATGTTC-3′, reverse: 5′ -CTCGCTCCTGGAAGATG-3′. PCR conditions were: 95°C for 3 min, followed by 40 cycles of 95°C for 10 s and 55°C for 30 s. Relative expression levels were calculated from the average threshold cycle number using the delta-delta Ct method.

### Putative G-quadruplex analyses

The QGRS mapper (http://bioinformatics.ramapo.edu/QGRS/index.php) was used to determine the potential G-quadruplex structures contained in genes of interest. Search parameters: maximal length: 45; minimal G-group size: 3; loop size: from 0 to 10 [7, 23].

### BG4 binding assay

BG4 antibody expression and purification was carried out as described for the HF2 antibody [56] with the following modifications. *E. coli* BL21 cells were transformed with the pSANG10-3F-BG4 plasmid. The expression of the BG4 single–chain antibody was then induced by 100 mM isopropyl β-D-1-thiogalacto-pyranosid. Cells were centrifuged and pellets were resuspended in a lysis buffer (25 mM Tris-HCl, 100 mM NaCl, 10% glycerol, 1% NP-40 and 10 mM imidazole) and sonicated using the QSONICA sonicator. Purification of the 6XHis-tagged BG4 antibody was carried out using HisPur Ni-NTA resin (Thermo Scientific). The eluted protein was concentrated with the Amicon Ultra-4 Centrifugal Filter and stored at −20°C in 50% glycerol. For the binding assay, 5′ - Cy5-labelled oligoes (Sigma) were re-suspended in 10 mM Tris-Cl containing 100 mM LiCl, NaCl, or KCl and denatured at 95°C for 5 min and then slowly cooled overnight to allow secondary structure formation. Annealed oligos were mixed with the purified BG4 antibody in 100 mM LiCl, NaCl or KCl, 20 mM HEPES pH 7.5, 0.01% NP40, 5% glycerol, 5 mM MgCl_2_, and incubated at room temperature for 15 min before running on a 10% non-denaturing TBE-polyacrylamide gel with 0.5X TBE. Gel images were captured using the BioRad Chemidoc imager. Sequences of the oligos are: BRCA1-G1: Cy5-AGGGCTCTCCCTTGGGGGGGGGGGGCAGGAAGGGA; BRCA1-G2: Cy5-AGGGAGGCCATGGGACGGGAAGACTTGGGT; BRCA1-cont: Cy5-AGCTTCCAAAATAGCCAACTGCGAGCTAATTCTGT.

### Statistical analyses

The statistical analyses and tests were performed with ImageJ software.

### Ethics statement

Rats were maintained in accordance with guidelines and regulations of the University of Texas McGovern Medical School (the protocol number is #AWC-16-0081). All experimental protocols were approved by the University of Texas McGovern Medical School. The methods were carried out in accordance with the approved guidelines.
